# Determinants of relative and absolute concentration indices: evidence from 26 European countries

**DOI:** 10.1186/1475-9276-12-53

**Published:** 2013-07-18

**Authors:** Tinna Laufey Ásgeirsdóttir, Dagný Ósk Ragnarsdóttir

**Affiliations:** 1 Department of Economics, University of Iceland, Oddi v/Sturlugotu 101, Reykjavik, Iceland

**Keywords:** Concentration index, Decomposition, Health-income inequalities, Health, Income, Net income, Gross income, Europe

## Abstract

**Introduction:**

The aim of publicly-provided health care is generally not only to produce health, but also to decrease variation in health by socio-economic status. The aim of this study is to measure to what extent this goal has been obtained in various European countries and evaluate the determinants of inequalities within countries, as well as cross-country patterns with regard to different cultural, institutional and social settings.

**Methods:**

The data utilized in this study provides information on 440,000 individuals in 26 European countries and stem from The European Union Statistics on Income and Living Conditions (EU-SILC) collected in 2007. As measures of income-related inequality in health both the relative concentration indices and the absolute concentration indices are calculated. Further, health inequality in each country is decomposed into individual-level determinants and cross-country comparisons are made to shed light on social and institutional determinants.

**Results:**

Income-related health inequality favoring the better-off is observed for all the 26 European countries. In terms of within-country determinants inequality is mainly explained by income, age, education, and activity status. However, the degree of inequality and contribution of each determinant to inequality varies considerably between countries. Aggregate bivariate linear regressions show that there is a positive association between health-income inequality in Europe and public expenditure on education. Furthermore, a negative relationship between health-income inequality and income inequality was found when individual employee cash income was used in the health-concentration measurement. Using that same income measure, health-income inequality was found to be higher in the Nordic countries than in other areas, but this result is sensitive to the income measure chosen.

**Conclusions:**

The findings indicate that institutional determinants partly explain income-related health inequalities across countries. The results are in accordance with previously published theories hypothesizing social mobility as the explanation for differences in health-income inequalities between countries and higher health-income inequality could be a result of lower income inequality.

## Introduction

The health-care industry is one of the world’s largest and fastest growing industries around the globe and can form an enormous part of a country’s economy. According to the Organization for Economic Co-operation and Development (OECD), health-care expenditures have grown faster than gross domestic product (GDP) in practically every country of the organization in the last decades [1]. How health systems work is obviously a major issue for those in need of health-care services. Less obvious, but equally important, is the economic performance of the system. A badly functioning system will demand a large proportion of available resources and deliver a low level of services. Given the size of the sector such a performance could seriously damage the well-being of the population. Thus, it is important to assess possible determinants of health or ill-health and its distribution, evaluate them in a critical manner, hold them up against each other, and compare across nations.

An evaluation of this kind is presented here. The aim of publicly-provided health care is twofold. First, some argue that health itself is a worthwhile production from a societal perspective due to externalities and its influence on economic growth. This does not only regard communicable diseases, but may also apply in general to human-capital formation. Living among healthy, well-educated and creative individuals is a source of happiness, which has been used as a rationale for subsidizing education, the arts, and health care. The second reason concerns equality. This view of entitlement has a different appeal regarding different goods, but health is one of the primary desiderata that many feel should not be a part of a society’s broad reward system. It is thus not simply the idea to decrease variation in health, but rather, to decrease variation in health by socio-economic status (SES).

Unlike the literature on income inequality, studies regarding inequalities in health are rarely concerned with pure inequality (unrelated to variations in any other variable). However, examples of this work do exist, using the Gini coefficient, and at times, the Atkinson’s index [2-4]. Those who feel that the socio-economic aspect of this question is important have criticized their approach, as it does not take into account whether persons in ill health are rich or poor [5].

Socio-economic inequalities in health have been widely studied across countries using different methods and different proxies for socio-economic status, such as income, education and employment. Measures that have been used include calculations of odds ratios, rate ratios and the relative index of inequality (RII). A study on eleven European countries examined socio-economic inequalities in health using mortality and self-reported morbidity as health measures, and income, education and occupational class as the socio-economic variables. Norway and Sweden were surprisingly found to have larger relative inequalities in health than most other countries and France as well when mortality was the health measure [6]. Another study on eleven European countries found similar results; a tendency for inequalities to be high in Norway, Sweden and Denmark [7]. These results are not in line with conventional beliefs, and indicate that health-income inequality is high in countries with more egalitarian welfare systems.

However, the most common measure of socio-economic-related health inequalities is the concentration index (CI). Examples of early work on the general health-income concentration includes Propper and Upward [8], who examined health inequalities by income using British data from the 1970s and 1980s. They employed equivalent household income and four different measures of health. Of the health variables used, the self-assessed health (SAH) showed the greatest income-related inequality in health. The other measures of health were: presence or absence of non-limiting chronic illness, the presence or absence of limiting chronic illness, and a dichotomization of SAH.

Van Doorslaer et al. [9] examined income-related inequalities in health for nine countries by calculating CIs. Individuals were ranked by equivalent household income, and health was measured by SAH. The study found pro-rich inequalities in health in all nine countries. The results showed the two Scandinavian countries within their cross-country analysis (Sweden and Finland) to possess relatively low income-related health inequality. The greatest income-related inequality was found in the United States, followed by the United Kingdom. The study revealed that in other countries income-related inequality in health was not as significant. Besides Sweden and Finland, those countries were East and West Germany, the Netherlands, Switzerland, and Spain. Those results were thus largely in accordance with conventional beliefs, showing Nordic countries with strong welfare systems to have relatively low inequality.

While including few countries, the study by van Doorslaer and his colleagues was of great importance for this literature and boosted research on health-income related inequality. It was an important project as it provided an excellent base that other researchers could compare their specific, national results to – and they did. The work of van Doorslaer and his colleagues thus provided an important base that could be used by other researchers. Humphries and van Doorslaer [10] examined income-related health inequality in Canada and Clarke and Smith [11] in Australia. They compared their results to van Doorslaer et al. [9] and found the level of inequality not to be significantly different from the United States or the United Kingdom but significantly greater than seven European countries. Ásgeirsdóttir [12] calculated the CI for Iceland and found income-related health inequality to be in accordance with findings from the Nordic countries in van Doorslaer et al. [9]. Again the cross-country comparisons appeared to be somewhat in accordance with conventional wisdom and with the results by van Doorslaer et al.

More studies have emerged that compare socio-economic inequality in health across countries. Van Doorslaer and Koolman [13] extended earlier work [9] in another study on health-income inequality in thirteen European countries by using an interval regression approach to compute CIs and by decomposing inequality into its determinants. Health-income inequalities favoring the rich were found for all countries and were high in Portugal, but also in the UK and Denmark, whereas they were low in the Netherlands and Germany, and also in Italy, Belgium, Spain, Austria and Ireland. The cross-country pattern of those results is thus not fully in accordance with previous findings from studies using CIs to measure health-income inequality or with conventional beliefs.

Other recent studies have examined health-income inequality with different methodological approaches. They have, like van Doorslaer and Koolman [13], found surprisingly extensive inequality in countries with welfare systems firmly rooted in egalitarian views, such as the Nordic countries [14-17]. A comparison study between Sweden and East and West Germany detected associations of similar magnitude between poor health and income in all regions [18]. Mackenbach et al. [14] followed up on previous work [6] and compared inequalities in self-assessed health and mortality among 22 countries in Europe. They found larger variability in the magnitude of inequalities in health than before. The highest socio-economic health inequality based on mortality was in Eastern Europe, but lowest in Southern Europe. When self-assessed health was the health measure largest inequalities were found in the Nordic countries and England. Another study compared health-income inequality in five different welfare regimes of Europe. The largest inequalities were found in the Anglo-Saxon welfare states (UK and Ireland) and the smallest in the Bismarckian regimes (Germany, France, Austria, Belgium and the Netherlands), while the inequalities were intermediate in the Scandinavian countries (Denmark, Finland, Norway and Sweden) [15]. Similar results were found when unemployment was used as the socio-economic variable [16], but highest inequalities were found in the Southern European welfare regimes when education was the socio-economic variable [17]. Huijts, Eikemo and Skalicka [19] examined differences in income-related health inequalities among the Nordic countries and found them to be highest in Norway and Finland but lowest in Denmark.

The reasons for such a cross-country pattern are not clear, if in fact it is found to be robust across methodological approaches and different data sources. Hypothesized reasons could be multiple. For example, it could be that low socio-economic status is more stressful in countries with more equality of opportunity and greater social mobility as it is more revealing of individual characteristics [20]. As those of higher socio-economic status could be more productive users of health-care services [21], and thus might get greater benefit from the provided services, such provision could potentially increase the health-income gradient. It has also been suggested that the bivariate measures used in the above-mentioned studies may, due to their design, be very sensitive to income inequality within a society [20]. Lastly, it could be that data collection among the lowest socio-economic status groups is more complete within certain societies, for example those with strong welfare regimes. But before such hypotheses are formally tested, it is valuable to get a good overview of the measured inequalities across countries and the within-country determinants of those inequalities. Here we propose such a descriptive representation of the data.

The relative CI does not take into account the level of health within the population, only how much it varies. The absolute concentration index (ACI), however, is a statistical measurement containing information on both the level and distribution of a variable. It is thus important as it captures information about the two main goals of publicly-provided health production simultaneously and is not sensitive to whether it is estimated with respect to health or morbidity [22]. The ACI is thus not simply an inequality measure but a broader goal-attainment measure, capturing the two main objectives of public health policy.

Only a few studies focusing on ACIs are available. Comparisons of socio-economic inequalities in health where both relative CIs and ACIs are measured have for example been made between Australia and England, and Australia and New Zealand [23,24]. Furthermore, a longitudinal analysis on fourteen member states of the European Union where both the relative CIs as well as ACIs were calculated has been made [25]. To the knowledge of the authors no other studies allowing for wide cross-country comparisons in ACI are available.

In this paper both CIs and ACIs will be calculated for 26 European countries with different cultural, social and institutional settings. Subsequently, the CIs will be decomposed and contributions of determinants to inequality in health calculated. Results will finally be used in cross-country comparisons. In the current study we thus address the following two questions: To what extent is ill-health concentrated among the poor within European communities? What are the individual and aggregate determinants of health and income-related health variations?

This study contributes to the literature in the following ways. First, we provide a broader range of countries for which CIs and ACIs are calculated than previously done, allowing for a more meaningful comparison across countries with different characteristics. Second, the robustness of previous results, showing for example high inequality in the Nordic countries, needs to be tested across data and evaluation methods. We thus provide an alternative analysis to the existing ones, using data on more countries than previously examined using CIs or other methods for that matter. This data was designed to be comparable across countries. Third, the study provides a renewed base to which others can compare their country-specific results. Fourth, we examine what factors contribute to inequalities and whether there are systematic differences between countries’ CIs and ACIs.

## Methods

The data utilized in this study provides information on 440,000 individuals in 26 European countries. The European Union Statistics on Income and Living Conditions (EU-SILC) is specifically aimed at providing data that are comparable between countries. It is anchored in the European Statistical System (ESS) and collected by Eurostat. EU-SILC was launched in 2004 in 13 European Union (EU) countries, but has since grown, and data collected in 2007 include 26 European countries. The countries included are: Austria (n = 13,391), Belgium (n = 12,322), Cyprus (n = 8,470), Czech Republic (n = 19,384), Denmark (n = 11,610), Estonia (n = 11,971), Finland (n = 21,773), France (n = 20,357), Germany (n = 26,291), Greece (n = 12,346), Hungary (n = 18,490), Iceland (n = 6,567), Ireland (n = 10,892), Italy (n = 44,629), Lithuania (n = 10,913), Luxembourg (n = 7,913), Latvia (n = 9,270), Netherlands (n = 19,623), Norway (n = 11,706), Poland (n = 34,888), Portugal (n = 9,947), Spain (n = 28,656), Sweden (n = 14,204), Slovenia (n = 24,730), Slovakia (n = 12,573), and the United Kingdom (n = 17,484).

The reference population of EU-SILC is all private households and their current members residing in the territory of the relevant countries at the time of data collection. Persons living in collective households and in institutions are generally excluded from the target population. This data contains information about income and socio-economic status, as well as health-related variables and detailed labor-market information. The data are collected from different sources and by different modes; constructed, deducted from sample frame, deducted from sample design, settled by interviewers, collected from household respondent, collected from household members or collected from a proxy. It is thus a complex dataset in terms of data origin. We will now discuss specifically the health and income variables used.

In a survey, the traditional five-level self-assessed health variable (SAH), ranging from “very good” to “very poor” was obtained. The literature shows that SAH predicts mortality and morbidity, even in the presence of additional controls [26-36]. Furthermore, this measurement is frequently used and will thus increase the chances for other researchers to calculate comparable health CIs for other countries that can be held up against the current estimates. In all instances, the numeric values of the SAH variable are such that a higher number indicates worse health, and thus ill-health CIs are calculated. Weighted average ill-health in the sample is 2.260 (SE 0.0024). When comparisons are made across countries it is important that the same health measure, SAH, is used when CIs and ACIs are calculated as previous results have shown estimates to be sensitive to the health measures used [8]. There are pros and cons to using the SAH variable in continuous form or dichotomized. Results in this study are qualitatively robust to such changes. However, especially when comparing other results to those reported here, it should be kept in mind that the reported results are those from estimations using the full information in SAH.

The main income measure used in the calculations is equivalized disposable household income, but calculations were made using individual gross employee cash or near cash income as well. Both of these measures were available for subjects of all countries, while most other income measures were only available for a few of the 26 countries and would thus have rendered cross-country comparisons less meaningful. What is furthermore important about those measures is that one measures the income rewards for individuals’ labor-market efforts, while the other one measures the resulting access to finances. This difference may be important, especially in countries with extensive income transfers, such as the Nordic countries. Individual gross employee cash or near cash income is the simpler of the two income measures. It includes the value of any social contributions and income taxes payable by an employee or by the employer on behalf of the employee to social insurance schemes or tax authorities [37]. It may be argued that equivalized disposable household income includes fuller information about individuals’ access to finances than individual gross employee cash income. It is constructed from total disposable household income multiplied with a within-household non-response inflator factor used to correct the effect of non-responding individuals within a household. Countries using the factor are Germany, Greece, Latvia, Portugal, Slovakia and Spain, while other countries imputed missing personal interviews. This multiple is then divided by equivalized household size which assigns the value 1 to an adult in a household, 0.5 to each additional household member aged 14 and over and 0.3 to each household member aged 13 or less. Equivalized disposable household income thus measures access to finances through own and family-members income, taking into account economies of scale in household production.

Total disposable household income is the sum for all household members of gross personal income components (gross employee cash or near cash income, gross non-cash employee income, gross cash benefits or losses from self-employment (including royalties), unemployment benefits, old-age benefits, survivor benefits, sickness benefits, disability benefits and education-related allowances), plus gross income components at household level including income from rental of a property or land, family/children related allowances, social exclusion not elsewhere classified, housing allowances, regular inter-household cash transfers received, interests, dividends, profit from capital investments in unincorporated business, income received by people under 16, minus regular taxes on wealth, regular inter-household cash transfer paid, tax on income and social insurance contributions [37]. However, some of these components are missing for a few countries (company car for France and Norway, regular taxes on wealth for Norway, sickness benefits for Italy, and non-cash employee income for the Netherlands), and therefore this measure is not entirely complete. However, those differences should not be expected to dramatically affect results. Income measures used were scaled to thousands of euros.

Other individual-level ill-health determinants used in the analyses are marital status, education, activity status, risk of poverty, as well as age and gender. Marital status dummy variables include married (reference category), divorced/separated, widowed and never married. Education levels are classified according to the International Standard Classification of Education (ISCED) and grouped into three categories: tertiary education (ISCED 5–6), upper secondary stage education or post-secondary non-tertiary education (ISCED 3–4), and lower secondary education or less (ISCED 0–2) (reference category). Activity status includes working full time (reference category), working part time, unemployed, student, retired, permanently disabled, in compulsory military community or service, fulfilling domestic tasks and care responsibilities, and other inactive. The risk of poverty threshold is set at household equivalized disposable income being less than 60% of its within-country median. All analyses are weighted using the cross-sectional personal weights provided within the EU-SILC.

Aggregate measures of income-related health inequalities are derived for the countries involved. The CI is based on the Lorenz curve, a cumulative frequency curve, which compares the distribution of a specific variable with the uniform distribution that represents equality. The ill-health-income concentration curve is a plot of the cumulative proportion of ill-health against the cumulative proportion of the population ranked by income. As such, it allows for examination of variations in one variable relative to variations in another variable. The income dimension is captured by the ranking of observations by income on the horizontal axis (with the least advantaged furthest to the left). The cumulative proportion of the ill-health variable is then represented on the vertical axis. The concentration curve can be compared with a diagonal line representing a uniform distribution, or perfect equality. The greater the deviation of the concentration curve from this line, the greater is the inequality.

The numeric representation that goes with the concentration curve is the CI or the concentration coefficient and corresponds to twice the area between the concentration curve and the diagonal line. The CI provides a measure of socio-economic inequality in health. It ranges from -1 to 1, with 0 representing perfect equality and -1 and 1 representing total inequality. The CI can be computed straightforwardly with individual-level data using a formula proposed by Kakwani, Wagstaff and van Doorslaer [38]:

$$2\sigma_{R}^{2}\left(\frac{y_{i}}{\mu }\right)=\alpha +\beta R_{i}+\epsilon_{i}$$

where *y*_*i*_ (*i* = 1, …, *n*) is the ill-health score of individual *i*, *μ* is the mean level of ill-health, *R*_*i*_ is the relative rank by income of individual *i*, *σ*_*R*_^2^ is the variance of *R*_*i*_, *β* is the CI, and *ϵ*_*i*_ is the error term.

The CI has a number of advantages as a measure of income inequalities in health. Most important, it reflects the experience of the entire population and not just those of two extreme socio-economic groups, as measures frequently used by non-economists do. The CI would thus change if the sizes of various groups changed, even if their mean health did not. One limitation of the CI is the fact that if everyone’s health were to double, the value of the index would not change. Such a difference would be captured in the ACI, which scales the relative CI by the mean of the health variable used [5]. That number would obviously increase if everyone’s health was enhanced and is thus a summary measure for ill-health and its distribution. That measure can thus be taken as an overall measure of the extent to which the overarching goals of a health care system have been reached.

The CI does not take into account the fact that demographics play a role in generating inequality in health. However, these factors can be taken into account by partitioning the CI into avoidable and unavoidable (age-gender) health inequality. Using the indirect method of standardization the inequality due to the age and gender composition of the sample can be computed and subtracted from the CI to obtain a standardized CI. This standardized CI then shows the health inequality that is potentially avoidable and thus relevant for policy purposes. Another approach, which is used here, is to decompose the relative CI into contributions of its various determinants, both unavoidable and avoidable, using the following linear regression model:

$$y_{i}=\alpha +\sum_{k}\beta_{k}x_{\mathrm{ki}}+\epsilon_{i}$$

where *y*_*i*_ is the ill-health measure for individual *i*, *x*_*ki*_ is an ill-health determinant for regressor *k* and individual *i* and *ϵ*_*i*_ is the error term. Given the relationship between *y*_*i*_ and *x*_*ki*_, the CI for *y* can be written as follows:

$$\mathrm{CI}=\sum_{k}\frac{\beta_{k}{\bar{x}}_{k}}{\mu }\;CI_{k}+\frac{\mathrm{GC}I_{\epsilon }}{\mu }$$

where *μ* is the mean of y, ${\bar{x}}_{k}$ is the mean of *x*_*k*_, *CI*_*k*_ is the CI for *x*_*k*_ and *GCI*_*ϵ*_ is the generalized CI for the error term. CI is thus equal to the sum of all the CIs of the k regressors weighted by the elasticity of y with respect to *x*_*k*_. *GCI*_*ϵ*_ is the residual and reflects health inequality not explained by systematic variation across income in the *x*_*k*_.

Finally results are compared across countries with regard to varying social, cultural, and institutional conditions. It is natural to start with public health expenditures (data missing for Greece, Ireland, Italy and the UK), as they are partially intended to mitigate the health-income relationship. Similarly, we focus on general income inequality, as it may affect health inequality directly or through the design of the CI [20]. A recent theoretical publication suggested that more equality in opportunity might cause greater health inequality [20], and thus we use public expenditures on education (data missing for Greece) as a proxy for equality of opportunity. Furthermore we use two measures of GDP, GDP expressed in euros per capita and GDP expressed in purchasing power standards (PPS) per capita, both scaled to million. All these aggregate measures come from Eurostat Statistics Database [39]. Finally we use dummy variables for neighboring countries that share similar social and cultural attributes. Countries are categorized into the following areas: Nordic countries (Denmark, Finland, Iceland, Norway and Sweden), Eastern Europe (Czech Republic, Estonia, Latvia, Lithuania, Hungary, Poland, Slovakia and Slovenia), North-Western Europe (Austria, Belgium, Netherlands, Luxembourg, Germany, Ireland and UK) and Southern Europe (Cyprus, Greece, Portugal, Spain, Italy and France). All associations are examined using bivariate linear regressions. We emphasize that those bivariate regressions are not intended to be direct tests, but rather as a way to organize the data and present the patterns within it. All data were analyzed with Stata 11.0 software [40].

## Results

Means of all variables used can be found in Table 1 for each country. For most countries individuals rate their health better in high income countries than low income countries, but there are some exceptions. Individuals in Greece and Cyprus do for example rate their health better than expected based on their mean incomes. The countries with both the highest mean equivalized disposable household income and individual gross employee cash income are Luxembourg, Iceland and Norway, and their average health is in all instances good. With the lowest mean incomes are the Eastern European countries which all report their health worse than the cross-country average.

**Table 1:** Means of variables

**Country**	**Ill-health**	**HH income**	**Income**	**Age**	**Male**	**Educ 2**	**Educ 3**	**Never married**	**Divorced**	**Widowed**	**Part-time**	**Unemployed**	**Student**	**Retired**	**Disabled**	**Military**	**Home**	**Inactive**	**Poverty**	**N**
**Austria**	2.0127	20.897	13.275	46.57	0.4835	0.5889	0.1587	0.3002	0.0889	0.0816	0.0990	0.0443	0.0597	0.2714	0.0043	0.0023	0.0900	0.0075	0.1144	13391
**Belgium**	2.0808	19.242	13.765	46.58	0.4873	0.3784	0.3219	0.2980	0.1035	0.0750	0.1220	0.0620	0.0815	0.2317	0.0323	0.0000	0.0720	0.0200	0.1474	12322
**Cyprus**	1.8971	18.725	10.529	43.26	0.4885	0.4000	0.2540	0.2694	0.0438	0.0509	0.0481	0.0291	0.1083	0.1654	0.0083	0.0152	0.0824	0.0182	0.1644	8470
**Czech Republic**	2.3357	6.231	3.526	45.24	0.4821	0.7152	0.1145	0.2658	0.0909	0.0841	0.0178	0.0679	0.0852	0.2349	0.0427	0.0000	0.0394	0.0011	0.0833	19384
**Denmark**	1.9825	25.299	22.536	46.99	0.4897	0.4151	0.2263	0.3417	0.0868	0.0756	0.0789	0.0291	0.1021	0.2319	0.0504	0.0009	0.0067	0.0129	0.1220	11610
**Estonia**	2.5678	5.321	4.002	45.49	0.4489	0.5120	0.2539	0.3518	0.1148	0.1114	0.0394	0.0318	0.0875	0.2167	0.0368	0.0013	0.0442	0.0003	0.1977	11971
**Finland**	2.2286	20.965	14.998	47.25	0.4846	0.4042	0.2785	0.3462	0.1103	0.0659	0.0640	0.0551	0.0828	0.2247	0.0600	0.0046	0.0277	0.0058	0.1360	21773
**France**	2.1313	18.805	11.595	46.71	0.4800	0.4136	0.2269	0.3527	0.0729	0.0768	0.0903	0.0543	0.0813	0.2767	0.0276	0.0000	0.0374	0.0145	0.1238	20357
**Germany**	2.3742	19.676	12.928	47.42	0.4864	0.5614	0.1993	0.2807	0.0877	0.0819	0.1335	0.0633	0.0856	0.2606	0.0155	0.0030	0.0528	0.0120	0.1546	26291
**Greece**	1.8130	12.108	6.161	46.88	0.4881	0.3496	0.1770	0.2733	0.0317	0.0830	0.0456	0.0541	0.0749	0.2193	0.0164	0.0036	0.1405	0.0085	0.1982	12346
**Hungary**	2.6448	4.479	2.649	45.76	0.4647	0.5581	0.1526	0.2669	0.0992	0.1173	0.0243	0.0567	0.0804	0.2339	0.0972	0.0000	0.0426	0.0093	0.1089	18490
**Iceland**	1.8549	34.509	29.585	43.47	0.5049	0.4110	0.2145	0.4405	0.0508	0.0350	0.0905	0.0072	0.1331	0.1126	0.0358	0.0000	0.0221	0.0122	0.0920	6567
**Ireland**	1.7193	26.230	15.164	42.66	0.4964	0.3420	0.2462	0.4108	0.0520	0.0689	0.1298	0.0532	0.1201	0.0873	0.0414	0.0000	0.1538	0.0099	0.1731	10892
**Italy**	2.3757	17.517	8.820	48.15	0.4813	0.3378	0.1085	0.2888	0.0376	0.0986	0.0534	0.0450	0.0690	0.2225	0.0146	0.0003	0.1415	0.0547	0.1879	44629
**Latvia**	2.7764	4.078	3.000	45.62	0.4462	0.5497	0.1766	0.2831	0.1489	0.1306	0.0373	0.0460	0.0756	0.2303	0.0210	0.0002	0.0456	0.0146	0.2143	9270
**Lithuania**	2.6400	3.987	2.878	45.02	0.4544	0.5330	0.2301	0.2399	0.0795	0.1094	0.0206	0.0408	0.0994	0.2211	0.0423	0.0009	0.0230	0.0135	0.1863	10913
**Luxembourg**	2.0343	35.006	24.395	45.44	0.4918	0.3706	0.2141	0.2744	0.0715	0.0653	0.0963	0.0270	0.0887	0.1474	0.0262	0.0000	0.1654	0.0031	0.1196	7913
**Netherlands**	2.1115	21.240	16.638	45.99	0.4921	0.3903	0.2568	0.3271	0.0691	0.0631	0.2309	0.0139	0.0811	0.1383	0.0405	0.0000	0.1059	0.0465	0.0921	19623
**Norway**	2.0722	30.703	23.934	46.07	0.4955	0.4461	0.2552	0.3744	0.1421	0.0088	0.0739	0.0184	0.0986	0.1818	0.0663	0.0102	0.0025	0.0174	0.1268	11706
**Poland**	2.4718	4.232	2.666	44.22	0.4762	0.6151	0.1428	0.2718	0.0427	0.1077	0.0413	0.0786	0.0976	0.2062	0.0700	0.0000	0.0266	0.0422	0.1592	34888
**Portugal**	2.7195	10.061	6.073	46.50	0.4783	0.1656	0.1182	0.2700	0.0405	0.0870	0.0537	0.0624	0.0694	0.2134	0.0151	0.0000	0.0684	0.0210	0.1762	9947
**Slovakia**	2.4499	4.432	2.513	44.26	0.4645	0.6695	0.1543	0.2924	0.0537	0.0950	0.0150	0.0518	0.1168	0.2596	0.0164	0.0000	0.0027	0.0182	0.0959	12573
**Slovenia**	2.5200	11.065	7.309	45.33	0.4870	0.5644	0.1615	0.3754	0.0446	0.0775	0.0151	0.0719	0.1202	0.2872	0.0036	0.0000	0.0180	0.0025	0.1149	24730
**Spain**	2.3018	13.721	8.567	45.84	0.4905	0.2231	0.2448	0.3130	0.0360	0.0741	0.0529	0.0616	0.0706	0.1526	0.0213	0.0000	0.1237	0.0510	0.1913	28656
**Sweden**	1.9498	20.157	16.179	47.10	0.4875	0.5059	0.2758	0.3916	0.1270	0.0622	0.1307	0.0275	0.0910	0.2281	0.0404	0.0007	0.0076	0.0075	0.1071	14204
**UK**	1.9510	25.134	18.013	46.72	0.4860	0.5474	0.2206	0.3155	0.0997	0.0715	0.1362	0.0206	0.0530	0.2275	0.0447	0.0000	0.0564	0.0087	0.1819	17484

Notes: *HH income* equivalized disposable household income. *Income* individual gross employee cash income.

Table 2 shows the marginal effects of each determinant on ill-health, where equivalized disposable household income is one of the explanatory variables. Every row represents one multiple linear regression. For nearly all countries there is a significant negative association between ill-health and income (not significant in Lithuania), secondary education (not significant in Norway), and tertiary education, which indicates that those with higher incomes as well as those with higher level of education report better health. Males generally report better health than females, but in most countries this association is not statistically significant. Not surprisingly health worsens with higher age. In most countries never married, divorced, and widowed people report their health worse than those who are married. This varies substantially between countries though and is not statistically significant in many instances. Being unemployed, retired or disabled is positively related to ill-health, and in many countries the same is true for doing housework or being economically inactive for other reasons. Working part-time is also statistically significantly positively related to ill-health in most countries while being a student is associated with better health in some countries. Being at risk of poverty is associated with worse health in most countries, but not significantly for many countries.

**Table 2:** Ill-health regression coefficients

**Country**	**HH income**	**Age**	**Male**	**Educ 2**	**Educ 3**	**Never married**	**Divorced**	**Widowed**	**Part-time**	**Unemployed**	**Student**	**Retired**	**Disabled**	**Military**	**Home**	**Inactive**	**Poverty**
**Austria**	**−0.0070**	**0.0246**	0.0344	**−0.2273**	**−0.3633**	0.0444	**0.1078**	0.0303	0.0068	**0.4991**	**−0.0999**	**0.2005**	**1.8378**	0.1053	**0.0917**	**0.4916**	0.0757
**Belgium**	**−0.0027**	**0.0148**	**−0.0920**	**−0.1030**	**−0.1859**	0.0516	**0.1321**	**0.1822**	−0.0021	**0.2617**	**−0.0950**	**0.1342**	**1.3367**		**0.1118**	0.1589	**0.1512**
**Cyprus**	**−0.0040**	**0.0261**	−0.0317	**−0.2342**	**−0.3834**	**0.0967**	**0.1692**	**0.1140**	**0.1711**	**0.2245**	0.0071	**0.3493**	**2.0484**	**0.0371**	**0.2304**	**0.5021**	**0.1160**
**Czech Republic**	**−0.0112**	**0.0286**	−0.0301	**−0.1401**	**−0.3437**	**0.0695**	0.0302	0.0446	**0.3992**	**0.3561**	**−0.1115**	**0.1752**	**1.3753**		−0.0337	**0.6248**	**0.0608**
**Denmark**	**−0.0019**	**0.0129**	−0.0298	**−0.1811**	**−0.2804**	**0.1050**	0.1091	0.1053	**0.0642**	**0.4974**	0.0069	**0.1556**	**1.4252**	0.7856	**0.6617**	**0.4015**	0.1134
**Estonia**	**−0.0094**	**0.0219**	0.0277	**−0.1186**	**−0.2459**	−0.0296	0.0488	0.0146	0.0740	**0.2574**	0.0031	**0.3523**	**1.2852**	0.0928	0.0526	0.0440	0.0196
**Finland**	**−0.0034**	**0.0207**	0.0241	**−0.1297**	**−0.2801**	**0.0544**	0.0226	−0.0002	**0.1135**	**0.3264**	0.0347	**0.1542**	**0.9816**	−0.1295	0.0549	0.2239	**0.0963**
**France**	**−0.0053**	**0.0231**	**−0.0531**	**−0.1269**	**−0.2440**	**0.0974**	**0.0861**	0.0684	0.0400	**0.2115**	−0.0474	**0.0896**	**0.7244**		0.0181	**0.5779**	0.0499
**Germany**	**−0.0042**	**0.0199**	−0.0195	**−0.1035**	**−0.1994**	0.0021	0.0239	−0.0287	0.0252	**0.3603**	**–0.1086**	**0.0916**	**1.3924**	**–0.1476**	0.0352	**0.3051**	**0.0526**
**Greece**	**–0.0042**	**0.0269**	**–0.0589**	**–0.1673**	**–0.2343**	**0.1343**	**0.1762**	**0.3050**	**0.0830**	**0.1555**	**0.1668**	**0.4609**	**2.2859**	**0.1892**	**0.1556**	**0.4238**	**0.0540**
**Hungary**	**–0.0280**	**0.0277**	**–0.0442**	**–0.1276**	**–0.2928**	**–0.0743**	**0.0307**	–0.0199	**0.2655**	**0.1397**	**−0.1621**	**0.2731**	**1.0420**		0.0080	**0.3513**	**0.0794**
**Iceland**	**−0.0020**	**0.0099**	−0.0282	**−0.1914**	**−0.3575**	0.0270	0.1347	−0.0226	**0.1776**	0.4067	−0.0221	**0.3660**	**1.5387**		**0.3423**	**1.2793**	−0.0088
**Ireland**	**−0.0027**	**0.0127**	0.0073	**−0.1170**	**−0.1453**	**0.1370**	**0.2718**	0.0337	**0.0787**	**0.1440**	0.0236	**0.2592**	**1.2413**		**0.2204**	**0.2471**	0.0746
**Italy**	**−0.0030**	**0.0231**	**−0.0690**	**−0.1250**	**−0.2248**	**0.0799**	**0.0568**	**0.0919**	**0.0565**	**0.1118**	0.0343	**0.1294**	**1.1872**	0.1332	**0.0592**	**0.2701**	0.0154
**Latvia**	**−0.0213**	**0.0210**	−0.0346	**−0.0813**	**−0.2182**	−0.0115	**−0.0659**	**0.0598**	0.0808	**0.1690**	−0.0764	**0.3353**	**1.3456**	**−0.3581**	0.0849	**0.4873**	**0.0560**
**Lithuania**	−0.0032	**0.0236**	**−0.0760**	**−0.0983**	**−0.2098**	0.0392	0.0561	0.0556	**0.2400**	**0.2031**	**−0.0805**	**0.3157**	**1.2015**	−0.2819	**0.1412**	0.1556	0.0291
**Luxembourg**	**−0.0029**	**0.0210**	−0.0524	**−0.1299**	**−0.2118**	**0.0951**	−0.0179	0.1044	−0.0300	**0.3484**	**−0.1977**	0.0626	**0.8496**		−0.0189	0.3374	**0.1746**
**Netherlands**	**−0.0021**	**0.0094**	−0.0122	**−0.1257**	**−0.2085**	**0.1389**	**0.1966**	**0.1000**	**0.0771**	**0.3107**	−0.0744	**0.1567**	**1.2031**		**0.3024**	**0.2433**	0.0552
**Norway**	**−0.0023**	**0.0083**	0.0146	−0.0663	**−0.2368**	0.0321	**0.1248**	−0.0555	**0.2683**	**0.3972**	−0.0454	**0.2894**	**1.2276**	**1.2339**	−0.2247	0.1654	−0.0273
**Poland**	**−0.0158**	**0.0288**	**−0.0675**	**−0.1253**	**−0.2620**	**0.0403**	**0.0667**	**−0.0669**	**0.1268**	**0.1187**	**−0.0939**	**0.2865**	**1.0794**		**0.0826**	**0.3010**	**0.0519**
**Portugal**	**−0.0078**	**0.0221**	**−0.1210**	**−0.1579**	**−0.2625**	0.0366	−0.0268	−0.0619	**0.1202**	**0.1293**	−0.0409	**0.3629**	**1.2841**		**0.1582**	**0.5098**	**0.1620**
**Slovakia**	**−0.0182**	**0.0311**	**−0.0386**	**−0.1601**	**−0.2995**	−0.0462	**0.1097**	−0.0086	**0.3515**	**0.1032**	**−0.1421**	**0.4369**	**1.6680**		**0.2916**	0.0625	0.0317
**Slovenia**	**−0.0147**	**0.0231**	−0.0067	**−0.2548**	**−0.4763**	0.0542	−0.0697	−0.0663	**0.5630**	**0.3231**	**−0.1139**	**0.2078**	**1.2304**		**0.2419**	**0.5421**	**0.0884**
**Spain**	**−0.0072**	**0.0202**	**−0.0594**	**−0.0953**	**−0.1313**	**0.0488**	0.0689	0.0457	0.0439	**0.1566**	0.0170	**0.1763**	**1.2617**		**0.1293**	**0.2944**	0.0114
**Sweden**	**−0.0070**	**0.0094**	−0.0259	**−0.1237**	**−0.2130**	0.0365	**0.0902**	−0.0082	**0.2411**	**0.3664**	−0.0667	**0.2118**	**1.3771**	**−0.5399**	0.0184	0.1599	0.0059
**UK**	**−0.0025**	**0.0093**	−0.0189	**−0.1369**	**−0.2414**	**0.0429**	**0.0846**	0.0227	**0.0449**	**0.1918**	**−0.0860**	**0.2527**	**1.5470**		**0.2235**	0.1233	−0.0086

Notes: *HH income* equivalized disposable household income. Regression coefficients which differ significantly from zero (at p < 0.05) are in bold typeface. Each row represents one linear regression.

First unstandardized CIs where equivalized disposable household income is the income measure were calculated and are reported in Table 3 for all countries. A negative sign indicates pro-rich inequalities while a positive sign indicates pro-poor inequalities. The results are all negative and statistically significant indicating that there is income-related health inequality in all 26 European countries favoring those with higher incomes. The degree of inequality varies considerably between countries and is highest in Cyprus while it is lowest in Poland. When calculating the ACIs the same countries remain with the highest and lowest deviations from health-policy goal attainment. However, the ranking of most countries changes slightly. ACIs show that most Eastern European countries have relatively high deviations from goal attainment, with the exceptions of Hungary and Poland. Most Nordic countries have lower deviations from goal attainment than average, with the exception of Finland. Austria and the Netherlands, and Ireland and the UK have ACIs around average, while Belgium has somewhat higher deviation from goal attainment and Germany and Luxembourg lower than the average.

**Table 3:** Concentration indices and absolute concentration indices using equivalized disposable household income

**Country**	**HH CI**	**HH ACI**	**HH Standardized CI**	**HH Standardized ACI**
**Austria**	−0.0506	−0.1018	−0.0484	−0.0974
**Belgium**	−0.0649	−0.1350	−0.0521	−0.1085
**Cyprus**	−0.1036	−0.1966	−0.0374	−0.0874
**Czech Republic**	−0.0542	−0.1266	−0.0732	−0.1389
**Denmark**	−0.0531	−0.1052	−0.0474	−0.0939
**Estonia**	−0.0673	−0.1729	−0.0437	−0.1121
**Finland**	−0.0585	−0.1304	−0.0484	−0.1079
**France**	−0.0359	−0.0764	−0.0380	−0.0810
**Germany**	−0.0383	−0.0910	−0.0319	−0.0758
**Greece**	−0.0575	−0.1043	−0.0471	−0.0854
**Hungary**	−0.0333	−0.0880	−0.0361	−0.0954
**Iceland**	−0.0503	−0.0933	−0.0480	−0.0891
**Ireland**	−0.0723	−0.1243	−0.0606	−0.1042
**Italy**	−0.0276	−0.0655	−0.0232	−0.0552
**Latvia**	−0.0516	−0.1433	−0.0355	−0.0984
**Lithuania**	−0.0479	−0.1264	−0.0289	−0.0762
**Luxembourg**	−0.0385	−0.0783	−0.0445	−0.0906
**Netherlands**	−0.0459	−0.0969	−0.0428	−0.0903
**Norway**	−0.0391	−0.0810	−0.0397	−0.0823
**Poland**	−0.0195	−0.0481	−0.0269	−0.0666
**Portugal**	−0.0523	−0.1423	−0.0427	−0.1161
**Slovakia**	−0.0445	−0.1089	−0.0303	−0.0742
**Slovenia**	−0.0632	−0.1593	−0.0544	−0.1371
**Spain**	−0.0446	−0.1026	−0.0313	−0.0720
**Sweden**	−0.0469	−0.0915	−0.0458	−0.0892
**UK**	−0.0595	−0.1160	−0.0511	−0.0997

Notes: *HH* equivalized disposable household income. *CI* concentration index. *ACI* absolute concentration index.

CIs for all countries were decomposed into their determinants. Avoidable inequality, i.e. health inequality not due to age and gender, was calculated from the decomposed CIs for each country. These age-gender standardized CIs are presented in Table 3 as well, along with the standardized ACIs. For most countries observed health inequality decreases when age and gender are adjusted for, with the exceptions of Poland, where it increases, as well as Hungary, Norway, and Sweden, where there is a slight increase. The standardized ACIs change in a similar way when age and gender are adjusted for, but also become worse for Luxembourg and France.

CIs and ACIs, both unstandardized and standardized, were also calculated using individual gross employee income as the income measure. Those results are reported in Table 4. All CIs are negative, but the results are quite different from those where equivalized disposable household income was the income measure. This difference between income measures chosen shows the effects of transfers in mitigating the health-income relationship as the equivalized disposable household income measure includes transfers while individual gross employee income excludes them. The results show a higher degree of inequality on average but less variation in health-income inequality, ranging from −0.0477 in Italy to −0.0945 in Iceland. Largest income-related health inequalities are in Nordic countries (Iceland, Denmark and Finland) and they are high in the other Nordic countries as well (Norway and Sweden). Most Eastern European countries have inequalities around average while lowest inequalities are mainly found in North-Western Europe and Southern Europe. The relative ordering of the countries changes substantially when ACIs are calculated. Slovenia has the highest deviation from goal attainment and most other Eastern European countries have above average deviation from goal attainment. Italy has the lowest deviation from goal attainment but other countries in Southern Europe have around average deviations from goal attainment and countries in North-Western Europe have low deviations from goal attainment.

**Table 4:** Concentration indices and absolute concentration indices using individual gross employee cash income

**Country**	**CI**	**ACI**	**Standardized CI**	**Standardized ACI**
**Austria**	−0.0838	−0.1686	−0.0393	−0.0792
**Belgium**	−0.0627	−0.1305	−0.0386	−0.0802
**Cyprus**	−0.0846	−0.1604	−0.0589	−0.1117
**Czech Republic**	−0.0706	−0.1649	−0.0414	−0.0968
**Denmark**	−0.0941	−0.1867	−0.0706	−0.1400
**Estonia**	−0.0709	−0.1820	−0.0471	−0.1211
**Finland**	−0.0933	−0.2078	−0.0604	−0.1346
**France**	−0.0712	−0.1517	−0.0313	−0.0667
**Germany**	−0.0497	−0.1179	−0.0234	−0.0556
**Greece**	−0.0864	−0.1566	−0.0485	−0.0878
**Hungary**	−0.0696	−0.1841	−0.0395	−0.1046
**Iceland**	−0.0945	−0.1752	−0.0847	−0.1571
**Ireland**	−0.0690	−0.1187	−0.0501	−0.0861
**Italy**	−0.0477	−0.1133	−0.0184	−0.0438
**Latvia**	−0.0633	−0.1757	−0.0380	−0.1054
**Lithuania**	−0.0606	−0.1600	−0.0357	−0.0942
**Luxembourg**	−0.0579	−0.1178	−0.0294	−0.0598
**Netherlands**	−0.0666	−0.1406	−0.0497	−0.1049
**Norway**	−0.0861	−0.1783	−0.0754	−0.1562
**Poland**	−0.0633	−0.1564	−0.0349	−0.0861
**Portugal**	−0.0628	−0.1709	−0.0364	−0.0990
**Slovakia**	−0.0685	−0.1679	−0.0404	−0.0990
**Slovenia**	−0.0843	−0.2123	−0.0487	−0.1227
**Spain**	−0.0619	−0.1425	−0.0312	−0.0718
**Sweden**	−0.0837	−0.1633	−0.0689	−0.1343
**UK**	−0.0683	−0.1333	−0.0528	−0.1030

Notes: *CI* concentration index. *ACI* absolute concentration index.

The standardized CIs and ACIs where individual gross employee income is the income measure are reported in Table 4 as well and show that both health-income inequality and deviations from goal attainment decrease in all countries when age and gender are accounted for. These results are very dissimilar to the results for standardized CIs and ACIs in Table 3 where equivalized disposable household income was the income measure. The Nordic countries have both the highest health-income inequality and the highest deviations from goal attainment, and Italy the lowest. Inequalities in Southern Europe are low, apart from Cyprus and Greece where it is above average. The standardized CIs show that the Eastern European countries have inequalities around and under average and their deviations from goal attainment are around average. In North-Western Europe health-income inequality ranges from above average to being one of the lowest, and the deviations from goal attainment in these countries show a similar pattern.

**Figure 1:**
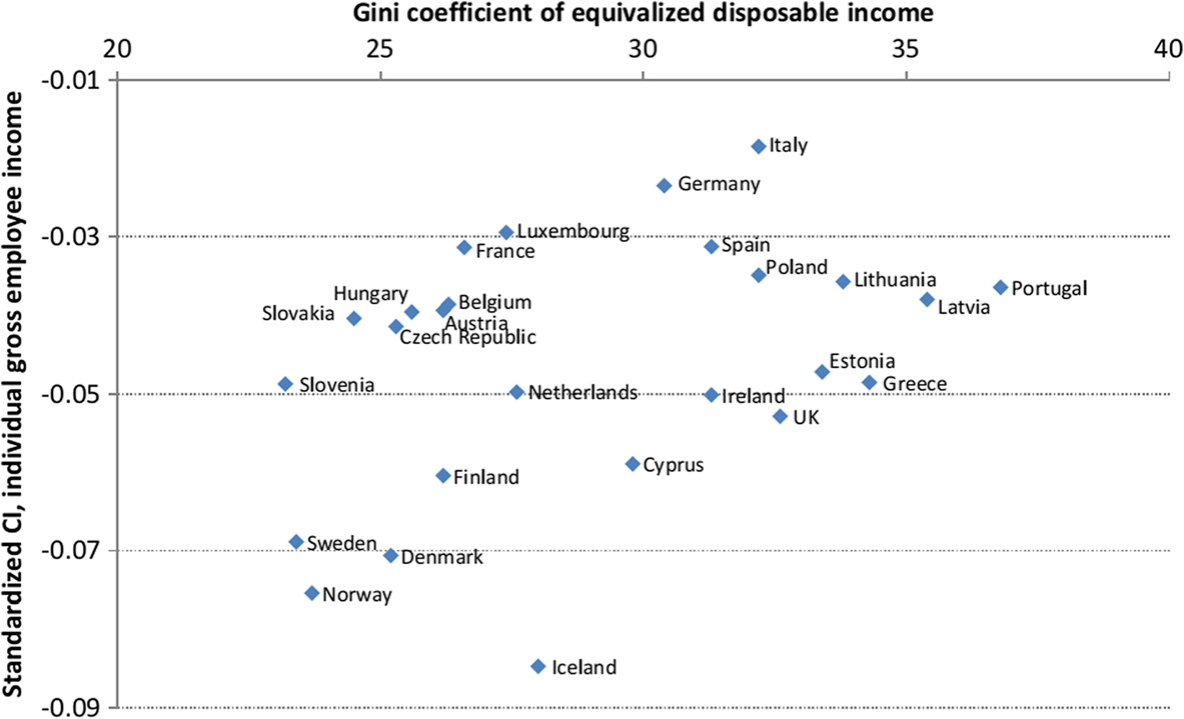
Standardized concentration index against Gini index.

**Figure 2:**
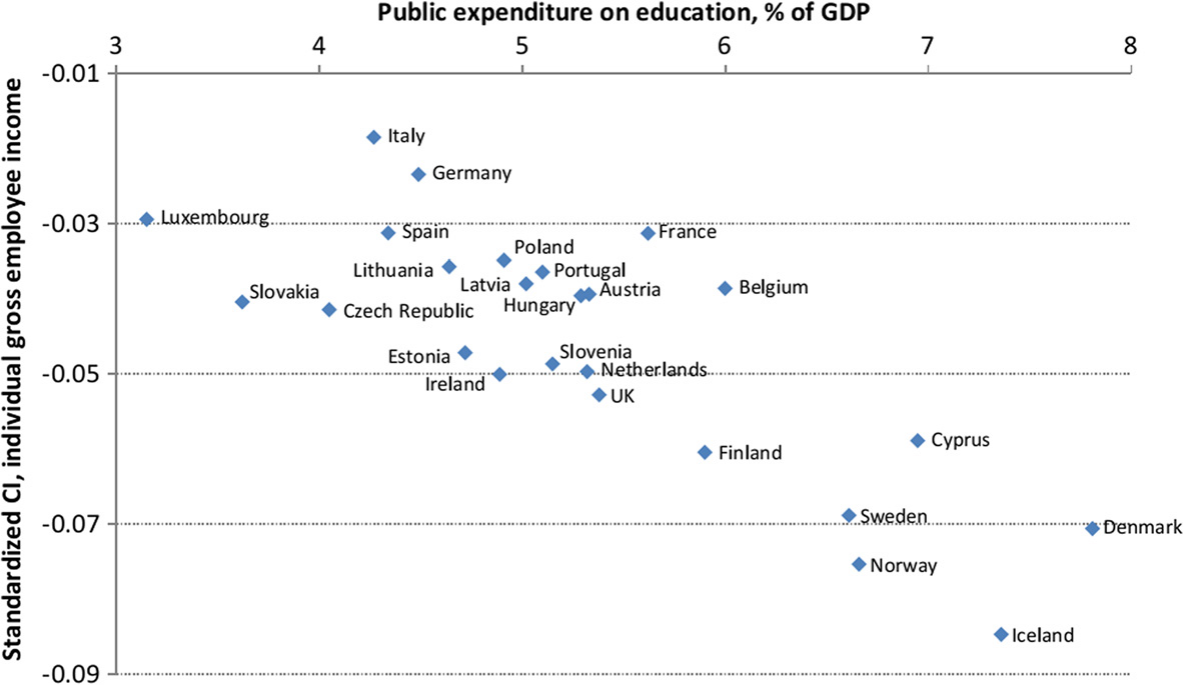
Standardized concentration index against public expenditures on education.

Bivariate linear regressions between aggregate measures as explanatory variables and CIs and ACIs, both unstandardized and standardized for age and gender, as the dependent variables are presented in Table 5. Each row represents four different bivariate linear regressions. These regressions help organize the data and make important patterns within it more apparent. The upper half of Table 5 shows results where CIs and ACIs with equivalized disposable household income as the income measure are the dependent variables. There is a statistically significant relationship between public expenditure on education and health-income inequality when standardized CI is the dependent variable and between public expenditure on health care and health-income inequality when ACI is the dependent variable. The lower half of Table 5 shows results where CIs and ACIs calculated with individual gross employee cash income are the dependent variables. Higher income inequality is statistically significantly associated with lower health-income inequality when CI is the dependent variable. However, as Figure 1 shows, this is mainly driven by the Nordic countries. Public expenditure on education has a negative statistically significant association with all the dependent variables. As can be seen in Figure 2, this is not simply driven by the Nordic countries, but shows a fairly systematic pattern across all the countries examined, although the Nordic countries certainly make up one end of the apparent pattern. There is a statistically significant relationship between the Nordic countries and all the dependent variables indicating higher health-income inequality in those countries compared to other areas. Furthermore, there is a negative association between Eastern Europe and ACI, and a positive association between North-Western Europe and ACI.

**Table 5:** Aggregate bivariate linear regressions

	**Dependent variables**				
	**HH CI**	**HH ACI**	**HH SCI**	**HH SACI**	**N**
Public expenditure on health care (% of GDP)	0.0036*	0.0108**	0.0004	0.0035	22
Gini index	−0.0003	−0.0015	0.0005	0.0004	26
Public expenditure on education (% of GDP)	−0.0048	−0.0038	−0.0050**	−0.0058	25
GDP PPS per capita (in millions)	0.0770	0.8780	−0.2560	0.0403	26
GDP euros per capita (in millions)	0.0515	0.6600*	−0.1910	0.0438	26
Nordic countries	0.0015	0.0142	−0.0040	0.0010	26
Eastern Europe	0.0045	−0.0143	0.0087*	−0.0002	26
Southern Europe	−0.0036	−0.0037	0.0001	0.0024	26
North-Western Europe	−0.0028	0.0076	−0.0064	−0.0027	26
	**Dependent variables**				
	**CI**	**ACI**	**SCI**	**SACI**	**N**
Public expenditure on health care (% of GDP)	−0.0012	0.0032	−0.0017	0.0001	22
Gini index	0.0015**	0.0020	0.0015*	0.0025*	26
Public expenditure on education (% of GDP)	−0.0088***	−0.0095**	−0.0116***	−0.0184***	25
GDP PPS per capita (in millions)	−0.0675	0.8760*	−0.2340	0.1310	26
GDP euros per capita (in millions)	−0.1630	0.4530	−0.3400*	−0.2120	26
Nordic countries	−0.0225***	−0.0286**	−0.0323***	−0.0549***	26
Eastern Europe	0.0047	−0.0235**	0.0075	−0.0053	26
Southern Europe	0.0039	0.0129	0.0110	0.0259*	26
North-Western Europe	0.0092	0.0365***	0.0074	0.0257*	26

Notes: *HH* equivalized disposable household income. *CI* concentration index. *ACI* absolute concentration index. *SCI* standardized concentration index. *SACI* standardized absolute concentration index. *GDP* gross domestic product. *PPS* purchasing power standard. Regression coefficients which differ significantly from zero (at p < 0.05) are in bold typeface. Each row represents four different bivariate linear regressions.

*p < 0.10 **p < 0.05 ***p < 0.01.

Mediation through education and activity status were examined by adding the % contributions of these determinants to the CIs to all the linear regressions. However, no mediation effects were found through these explanatory variables when examined in this way.

The decompositions of the CIs where equivalized disposable household income is the income measure are reported in Table 6. For each country each column shows percentage contributions from each determinant to overall income-related health inequality. The contributions depend on both the elasticity of ill-health with respect to the determinants and the income-related CI of each determinant. The largest contributions are income, age, education, and activity status. Income explains from 3.42% of total inequality in Lithuania to 44.33% in Poland. In most countries age contributes to inequality in health, with contributions ranging from 2.02% in Sweden to 38.31% in Lithuania, but in France, Hungary, Luxembourg, Poland, and slightly in Norway age reduces inequality in health. Secondary education contributes less than 5% to the CIs in all countries except for Italy (7.79%). However, tertiary education contributes considerably to health inequality or from around 10% (Estonia, Germany, Ireland, Latvia, Spain and Sweden) up to almost 40% in Poland. The total contribution of activity status ranges from 10.89% in Luxembourg to 41.20% in the UK. The largest contributions to inequality from activity status come from those who are unemployed, retired or disabled. In Austria, Czech Republic, Germany, and Slovenia the main contributions come from the unemployed, from 6.24% in Slovenia to 11.98% in Germany. The disabled are the largest contribution from activity status in most countries, ranging from around 5% in Portugal, Spain, and Italy to 30.96% in Poland. The contributions to inequality are from 11.53% (Cyprus) to 20.08% (Slovakia) in countries where the retired are the largest contribution. In most countries the risk of poverty contributes less than 10% to the unequal distribution of ill-health favoring the better off, except for 23.49% in Luxembourg and from 11.55% to 16.53% in Denmark, Belgium, Poland and Portugal. Gender and marital status both contribute to less than 5% of inequality in health and slightly decrease inequality in some countries.

**Table 6:** Percentage contributions to concentration indices using equivalized disposable household income

**Country**	**HH income**	**Age**	**Male**	**Educ 2**	**Educ 3**	**Never married**	**Divorced**	**Widowed**	**Part-time**	**Unemployed**	**Student**	**Retired**	**Disabled**	**Military**	**Home**	**Inactive**	**Poverty**
	**% contribution to concentration indices**																
**Austria**	37.29	5.03	−0.67	4.58	15.20	−0.23	1.10	0.33	0.00	9.35	−0.56	2.22	1.80	−0.01	2.18	1.51	7.53
**Belgium**	10.28	18.41	1.23	−0.98	13.20	−0.37	1.31	2.93	0.02	5.43	−0.60	4.24	10.89	0.00	1.94	0.68	14.08
**Cyprus**	11.86	29.05	0.29	1.58	17.82	−0.90	0.80	1.32	0.13	0.91	−0.04	11.53	3.15	−0.01	1.98	1.04	8.11
**Czech Republic**	13.77	30.48	0.50	0.67	12.12	−0.50	0.38	0.85	−0.06	9.96	−0.63	7.91	9.92	0.00	−0.17	0.12	3.67
**Denmark**	11.57	10.24	0.48	1.11	17.71	3.34	0.68	2.98	−0.52	4.63	0.22	10.37	12.24	0.08	0.55	0.41	11.55
**Estonia**	9.76	35.57	−0.41	−0.48	8.95	0.31	0.46	0.38	−0.08	2.02	0.01	16.78	13.22	0.02	0.08	0.01	1.79
**Finland**	14.43	17.62	−0.36	−2.22	20.18	1.07	0.21	0.00	0.41	5.44	0.47	7.25	10.97	−0.05	0.18	0.04	8.68
**France**	34.56	−6.87	0.93	−0.30	21.76	2.32	0.75	1.07	0.34	4.72	−0.78	0.59	7.41	0.00	0.27	3.79	7.08
**Germany**	26.51	16.35	0.37	−0.46	10.66	0.00	0.44	−0.38	−0.21	11.98	−0.77	3.84	7.96	0.02	0.24	1.06	7.55
**Greece**	16.54	17.59	0.55	2.92	16.37	−1.24	−0.15	3.50	0.51	1.96	1.12	6.11	11.03	−0.04	3.15	0.28	8.23
**Hungary**	36.55	−8.87	0.48	0.96	23.95	−0.40	0.40	−0.25	0.74	4.01	−2.28	0.09	23.12	0.00	0.11	1.39	8.75
**Iceland**	20.47	4.12	0.45	−1.65	19.86	0.56	1.81	−0.34	−0.47	0.24	−0.22	16.33	16.61	0.00	0.66	2.00	−0.79
**Ireland**	17.70	16.25	−0.08	1.22	10.62	0.83	2.88	0.54	0.07	2.34	0.33	4.09	18.21	0.00	7.75	0.57	8.59
**Italy**	25.82	13.78	2.03	7.79	16.81	−0.59	0.00	1.84	−0.13	2.79	0.27	0.85	5.37	0.02	3.50	5.27	3.59
**Latvia**	21.51	30.77	0.56	0.43	8.98	0.01	−0.64	1.48	0.37	1.74	−0.33	15.04	9.14	−0.02	0.44	0.87	6.58
**Lithuania**	3.42	38.31	1.42	−1.29	15.04	0.23	0.69	1.76	0.60	2.39	−0.40	17.05	11.20	−0.07	1.00	0.65	3.49
**Luxembourg**	34.89	−16.36	0.68	1.10	24.29	0.18	−0.18	−0.36	−0.05	5.65	−1.80	−0.25	7.46	0.00	−0.55	0.43	23.49
**Netherlands**	12.83	6.53	0.23	−0.42	16.80	−0.34	2.77	1.10	−2.05	1.19	−1.39	2.54	17.46	0.00	9.19	2.51	4.76
**Norway**	21.69	−1.17	−0.40	−0.14	18.55	1.77	4.98	−0.10	−1.72	3.58	−1.94	17.71	17.29	3.71	−0.18	1.02	−3.73
**Poland**	44.33	−39.03	0.62	−3.88	39.30	1.49	0.68	−1.38	0.68	7.50	−2.01	−9.91	30.96	0.00	0.78	5.33	14.45
**Portugal**	20.29	17.39	1.00	3.81	12.21	0.01	−0.01	−0.83	0.76	1.12	0.06	5.03	4.57	0.00	2.24	2.25	16.53
**Slovakia**	18.10	31.09	0.80	1.08	13.69	0.11	0.99	−0.20	−0.30	2.26	−2.04	20.08	4.90	0.00	0.17	0.37	2.53
**Slovenia**	23.51	13.89	0.06	−0.05	24.11	−0.06	−0.42	−0.94	0.31	6.24	−0.04	4.09	1.15	0.00	1.14	0.30	5.63
**Spain**	29.99	29.11	0.74	1.08	10.28	−0.52	0.13	0.61	0.04	2.24	0.08	3.33	5.18	0.00	3.98	3.74	1.72
**Sweden**	36.70	2.02	0.47	−0.65	10.37	0.95	1.73	−0.24	−1.65	3.23	−2.00	12.78	10.04	−0.02	0.03	0.30	0.61
**UK**	17.85	13.75	0.27	1.00	15.15	−0.07	0.89	0.46	−0.13	1.85	−0.70	14.15	22.21	0.00	3.57	0.24	−1.10

Notes: *HH income* equivalized disposable household income.

The decomposition results from CIs where individual gross employee income was the income measure are reported in Table 7. Using this income measure the largest contribution to health inequality favoring the rich is age in most countries, from 9.83% in Iceland to 58.73% in Italy. Individual gross employee income contributes from only 1.19% in Greece to 27.93% in Sweden. Other large contributors in most countries are tertiary education, being retired, and being disabled. The contribution of tertiary education ranges from 6.15% in Austria to 19.91% in Cyprus, whereas secondary education contributes to less than 6% in all countries. Being retired contributes from around 5-6% (Finland, France and Germany) to 27.60% (Slovakia) to inequality except for in Luxembourg where it lowers health inequality slightly. Being disabled contributes from almost nothing (Slovenia and Austria) up to 25.77% in Norway. In most countries the risk of poverty contributes less than 6% to the unequal distribution of ill-health favoring the better off, except for in Ireland (6.27%), Portugal (6.89%) and Belgium (7.45%). Marital status and gender contribute to less than 5% of inequality in health and slightly decrease inequality in some countries.

**Table 7:** Percent contributions to concentration indices using individual gross employee cash income

**Country**	**Income**	**Age**	**Male**	**Educ 2**	**Educ 3**	**Never married**	**Divorced**	**Widowed**	**Part-time**	**Unempl**	**Student**	**Retired**	**Disabled**	**Military**	**Home**	**Inactive**	**Poverty**
	**% contribution to concentration indices**																
**Austria**	12.68	54.78	−1.74	5.78	6.15	−0.54	−0.37	0.80	0.05	2.05	−2.13	12.69	2.01	−0.01	1.58	0.71	3.30
**Belgium**	6.67	35.51	3.00	0.34	11.83	−0.42	−0.57	4.66	0.17	3.74	−2.90	9.41	13.42	0.00	2.53	0.61	7.45
**Cyprus**	6.89	29.43	0.94	1.59	19.91	1.68	−0.13	1.63	0.64	0.32	−0.84	17.64	5.22	0.01	5.53	2.03	5.97
**Czech Republic**	16.82	40.83	0.51	2.04	6.84	0.05	−0.09	0.96	−0.24	4.05	−3.84	8.36	15.88	0.00	−0.88	0.14	1.36
**Denmark**	14.69	24.46	0.56	2.99	9.01	−0.47	0.15	2.47	−0.18	1.93	−0.63	7.21	19.95	−0.01	1.10	0.40	3.21
**Estonia**	9.59	34.75	−1.26	1.48	8.78	0.03	−0.21	0.49	0.03	1.13	−1.03	22.24	13.45	0.02	0.24	0.00	1.90
**Finland**	15.97	35.75	−0.53	0.77	11.74	−0.16	0.02	0.14	−0.09	2.09	−0.19	5.40	13.21	−0.14	0.00	0.07	3.17
**France**	8.93	54.91	1.15	1.70	11.42	−1.70	−0.17	1.77	−0.42	1.22	−1.80	5.51	6.36	0.00	−0.03	2.24	2.55
**Germany**	16.10	52.33	0.52	2.02	7.33	0.04	−0.12	−0.80	0.00	5.50	−3.92	5.59	8.26	0.02	−0.11	1.32	4.17
**Greece**	1.19	42.47	1.43	2.84	8.52	−1.05	−0.63	4.92	−0.37	0.43	2.56	20.27	7.54	0.09	4.79	0.64	2.28
**Hungary**	10.37	42.52	0.67	2.56	8.88	0.16	−0.15	−0.27	−0.52	0.40	−4.15	13.14	23.19	0.00	−0.14	0.61	2.18
**Iceland**	16.71	9.83	0.53	0.59	14.20	0.10	−0.10	−0.39	1.00	0.19	−1.84	15.19	22.02	0.00	1.71	2.18	0.08
**Ireland**	12.12	27.65	−0.18	1.51	10.42	−0.53	0.30	0.77	−1.07	1.58	0.12	8.35	19.96	0.00	12.59	0.57	6.27
**Italy**	4.85	58.73	2.62	5.18	6.62	−0.75	−0.34	2.58	−0.60	0.67	0.50	8.12	4.50	0.01	2.49	3.81	1.92
**Latvia**	14.33	39.22	0.78	1.45	7.48	−0.06	0.43	1.77	0.15	0.68	−2.26	22.21	8.01	−0.03	0.54	1.03	5.05
**Lithuania**	6.38	39.37	1.76	0.87	11.06	0.76	−0.09	1.63	0.13	1.06	−3.00	21.39	15.35	−0.05	0.83	0.50	1.42
**Luxembourg**	22.22	46.94	2.28	1.78	12.81	0.09	0.10	2.43	0.75	1.88	−9.78	−0.42	8.39	0.00	−5.00	0.32	5.62
**Netherlands**	8.98	24.94	0.42	2.03	10.26	−2.87	0.79	2.36	−1.87	0.85	−1.23	7.20	17.56	0.00	11.50	3.78	1.79
**Norway**	20.90	13.26	−0.84	0.32	9.11	−0.04	2.67	0.02	−0.19	1.04	−1.83	12.83	25.77	3.31	−0.07	0.42	−0.15
**Poland**	7.84	43.38	1.53	1.77	10.78	0.29	−0.04	−1.36	−0.30	1.23	−3.01	13.22	18.28	0.00	0.36	2.81	2.11
**Portugal**	7.22	39.73	2.34	1.31	7.69	0.06	0.03	−0.92	0.45	0.74	−1.36	18.31	4.46	0.00	2.42	1.97	6.89
**Slovakia**	18.37	40.27	0.79	3.00	8.08	−0.57	−0.22	−0.08	−0.25	0.57	−6.34	27.60	7.16	0.00	0.17	0.05	1.17
**Slovenia**	9.31	42.15	0.08	1.32	14.84	−0.72	−0.01	−0.95	−0.70	3.69	−1.66	11.94	1.05	0.00	0.96	0.20	3.67
**Spain**	7.94	47.57	2.03	0.80	8.52	−0.49	−0.21	1.00	−0.24	0.77	0.00	8.20	7.98	0.00	4.96	4.48	3.20
**Sweden**	27.93	17.51	0.26	1.10	6.85	−0.66	0.42	0.32	−1.21	1.41	−2.46	9.98	18.08	−0.05	−0.01	0.12	1.08
**UK**	12.03	22.34	0.39	2.29	10.89	−0.38	−0.17	0.66	−0.18	1.34	−1.82	20.40	25.45	0.00	4.01	0.25	1.36

Notes: *Income* individual gross employee cash income.

## Discussion

The results of the current study add to the existing knowledge on income-related disparities in health in Europe. Total CIs for ill-health are negative in all countries, implying that worse health is concentrated among those with lower income in Europe. Previous results, showing extensive income-related health inequality in the Nordic countries [6,7,13-17] are partially confirmed, although they appear to be sensitive to the income measure used in the analysis. The puzzling results for the Nordic welfare states are certainly found when income is measured with gross labor-market income, but disappear when transfers are accounted for. This is indication that the extensive transfer systems in the Nordic countries serve to mitigate the health-income relationship to a considerable extent. Cross-country comparison shows that both the highest income-related inequalities in health and the highest deviations from goal attainment when individual gross employee cash income is the income measure are in the Nordic countries and that they are statistically significantly higher than in other areas in Europe, while the results are more mixed when equivalized disposable household income is the income measure. The results show that the largest contributions to inequality within countries are income, age, education, and activity status. However, the magnitude of the contribution of each of these determinants varies considerably between countries, with some countries achieving a much higher degree of inequality than others, as has been found in Europe before [9,13]. Cross-country patterns in the contribution of different determinants of CIs and ACIs is limited.

The current study has both strengths and limitations. The dataset used in the analyses is large and contains a great amount of information about individuals in 26 European countries and therefore provides a good base for cross-country comparison. By calculating ACIs rather than only relative CIs as has been done in most previous studies we get information on both the level of a variable and its distribution. This is important as the aim of health policy is generally twofold, that is to produce health and attain health equality. The ACI thus provides a performance measure for the overarching aim of health policy.

Self-assessed health is used as an indicator of ill-health and has proven to be a good and valid indicator of health. However, there has been some concern about to what degree self-assessed measures are comparable across socio-economic groups. These measures may suffer from reporting bias since different people might not rate health in the same way, and thus income-related health inequality might be biased [41,42]. Another limitation is that the study’s cross-sectional design does not allow us to interpret any causal relationships. The results should thus be read as a description of the distribution of health. Other factors not included in the decomposition analyses, such as health-system related determinants, could also contribute to health inequalities.

The findings reported here, on income-related inequalities in health and their differences across Europe, provide evidence for better health among those who are wealthier within each society as well as across societies. To what degree within-country inequalities exist varies considerably between countries. Although better health is concentrated among the well-off within every country in Europe, these inequalities exist in low-income countries and high-income countries, as well as across countries with different levels of average health. There is limited indication that public health expenditures mitigate the health income relationship. Negative associations are found between ill-health CIs and ACIs, and public expenditure on education. Countries with high income-related health inequality tend to be the same countries as spend the most on education. This supports the idea that social mobility might be the explanation for high income-related health inequality in the Nordic countries. Furthermore, the Gini index is positively associated with ill-health CI in one estimation. Countries with high health-income inequality tend to have lower Gini indices, although this may apply to the Nordic countries in particular, but to a lesser extent to variations across other European countries. There may be a tradeoff between income and health inequality but the CI is not sensitive to health transfers and therefore this tradeoff might not be captured by the CI. Thus, it is possible that higher health-income inequality might be an indicator of lower income inequality [20].

The production of health and the mitigation of the health-income relationship has been the focus of large-scale government expenditures in many countries. The current study shows where potentials for reduction in inequality lie, but how this goal is best obtained is a question still to be pondered and the scale of the expenditures involved, leaves it a fiscally important subject.

## Competing interests

The authors declare that they have no competing interests.

## Authors’ contributions

Both authors collaborated on all tasks and sections of the paper. Both authors read and approved the final manuscript.
